# Increasing Incidence Rate of Cervical Cerclage in Pregnancy in Australia: A Population-Based Study

**DOI:** 10.3390/healthcare4030068

**Published:** 2016-09-12

**Authors:** Corrine Lu, Boon Lim, Stephen J. Robson

**Affiliations:** 1 Centenary Hospital for Women and Children, Canberra Hospital, Garran ACT 2605, Australia; Boon.Lim@act.gov.au (B.L.); stephen.robson@anu.edu.au (S.J.R.); 2 Australian National University Medical School, Canberra Hospital Campus, Garran ACT 2605, Australia

**Keywords:** cerclage, preterm, pregnancy, birth, population, incidence

## Abstract

Objective: Data published from the United States have demonstrated that the use of cervical cerclage has fallen in the period 1998–2013. This is in contrast to recommendations in Australia. We examined this trend using data from the Australian Institute of Health and Welfare (AIHW). Study design: Retrospective population-based study. Methods: Data from the Australian Institute of Health and Welfare procedural database were used to determine the total number of cervical cerclage sutures inserted during the period 2004 to 2013. Population datasets were used to calculate age-stratified incidence rates of cerclage. Findings: There was a significant increase in the rate of cervical cerclage in women aged 25 to 34 years and in the 35 years and older age group. The incidence of preterm birth was stable for gestations of 32 to 36 weeks, but slightly increased in the 20 to 27 week and 28 to 31 week gestational age groups. Further research into cervical cerclage and the use of vaginal progesterone for the prevention of preterm birth would be valuable.

## 1. Introduction

Preterm birth is the leading cause of perinatal death, complicating more than 10% of births internationally [1,2]. In addition to perinatal death, preterm birth is also the cause of both short- and long-term morbidity and disability. Despite a concerted international commitment to reducing the incidence of preterm birth, there is little evidence of any decrease: preterm birth rates may in fact have increased [3]. One strategy employed to reduce the risk of preterm delivery is the placement of a cervical cerclage suture (cerclage), in an attempt to prevent cervical dilatation and subsequent preterm birth. The first description of this procedure was published 60 years ago [4] and although it has undergone modifications [5] since that then, the technique for placement in pregnancy has changed little. Typically it is employed in three circumstances: where there is a history of sequential mid-trimester losses; when a diagnosis of cervical shortening is made on ultrasound examination during pregnancy; and, when cervical shortening, dilatation, or both are found on examination [6].

There is evidence that cerclage is superior to no treatment in the prevention of preterm birth and that its use might be associated with a reduction in the risk of perinatal death [6]. However there are higher rates of febrile and infectious morbidity and an increased rate of caesarean delivery in pregnancies managed with the use of cerclage. An evolving evidence base for the use of progesterone supplementation for the prevention of preterm birth has made the place of cerclage in pregnancy less clear. At the time of writing, there are no published studies of direct comparison of progesterone with cerclage in the setting of cervical shortening. Indirect comparisons made by meta-analysis have not shown one method to be superior [7]. Despite this continuing uncertainty, recent data from the United States have suggested that cerclage use has fallen by more than half over that last 16 years [8]. The Royal Australian and New Zealand College of Obstetricians and Gynaecologists (RANZCOG) has endorsed a recommendation for consideration of cerclage since 2008 [9], thus, we set out to determine whether a similar trend in the use of cerclage was evident at a national level in Australia.

## 2. Methods

Data regarding pregnancy-associated cervical cerclage were obtained from the Australian Institute of Health and Welfare (AIHW) national procedural dataset, collected through the Australian National Health Information Agreement under the auspice of the Australian Health Ministers’ Advisory Council (AHMAC). This is specified in the National Minimum Data Sets relating to hospitals and day procedure facilities, and comprises pooled data supplied by Australian state and territory health authorities. It provides information about procedures performed on admitted patients in hospitals and day surgery facilities. To ensure no pre-pregnancy (interval) cerclage data were included (either laparoscopic or transvaginal), data was extracted based on the ICD-10-AM (Australian Modification) in the procedure chapter XIII, using the ICD specific codes 16511-00 (insertion of cervical suture). The data was collected in age bands and extracted to an Excel™ spreadsheet for analysis. To obtain denominators, the total numbers of term and preterm births for each year of the study were obtained from the AIHW Australia’s Mothers and Babies series [10]. Linear regressions were performed to calculate R and adjusted R^2^ (aR^2^) values, and *p*-values. This study received prospective approval from the Australian National University Human Research Ethics Committee (protocol 2015/347).

## 3. Results

**Figure 1:**
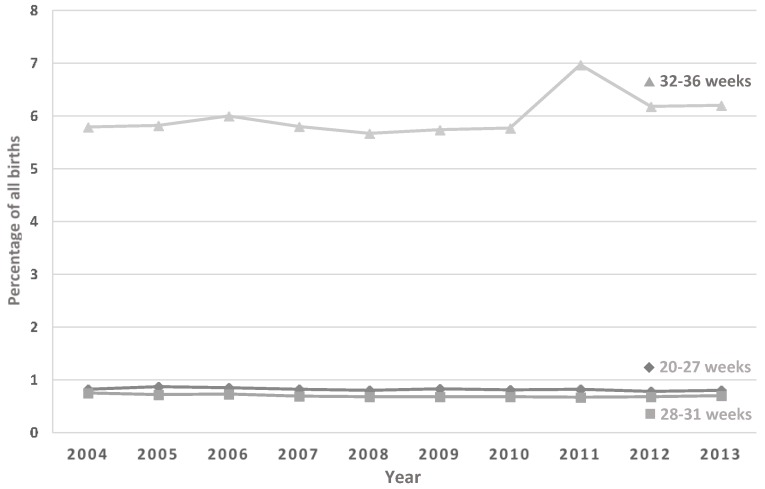
Preterm births as a percentage of all births in Australia, 2004 to 2013. There have been slight but significant increases in the percentage of preterm births in the 20–27 week group (R = 0.68; aR^2^ = 0.47; *p* = 0.03) and 28–31 week group (R = 0.73; aR^2^ = 0.48; *p* = 0.02) but no change in the 32–36 week group (R = 0.52; aR^2^ = 0.18; *p* = 0.12). From AIHW Australia’s Mothers and Babies series.

**Figure 2:**
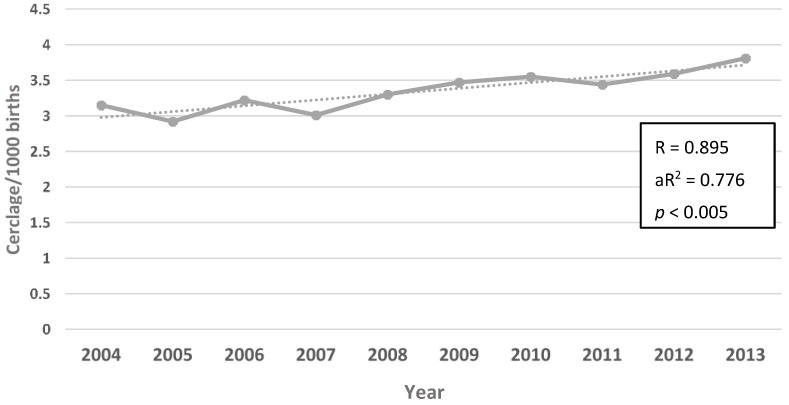
Overall incidence rate of cervical cerclage (procedures per 1000 births) in Australia for the period 2004 until 2013.

**Figure 3:**
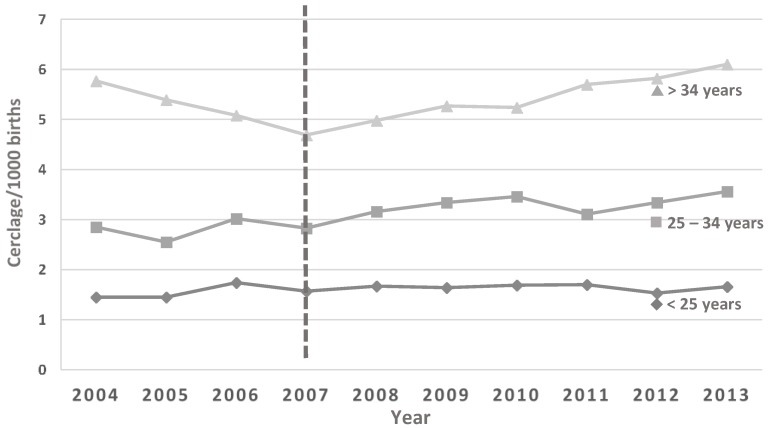
Maternal age-stratified incidence rates for cervical cerclage (procedures per 1000 births) in Australia for the period 2004 until 2013. There was no increase in the rate of cerclage in the age group less than 25 years across the study period (R = 0.46; aR^2^ = 0.11; *p* = 0.19), but significant increases since 2007 in the 25 to 34 year age group (R = 0.84; aR^2^ = 0.67; *p* = 0.002) and the 35 years and older age group (R = 0.99; aR^2^ = 0.96; *p* < 0.005).

Data were obtained for the period from July 2004 to June 2013. Over that period, there was a slight but significant increase in the percentage of preterm births in both the 20 to 27 week group (R = 0.68; aR^2^ = 0.47; *p* = 0.03) and the 28 to 31 week group (R = 0.73; aR^2^ = 0.48; *p* = 0.02), but no observed change in the 32–36 week group (R = 0.52; aR^2^ = 0.18; *p* = 0.12) (Figure 1). The incidence rate of cerclage (procedures per 1000 births) increased significantly, from just over 3/1000 births to 3.8/1000 births (R = 0.895; aR^2^ = 0.776; *p* < 0.005) over the study period (Figure 2). When the incidence rates of cerclage were stratified by maternal age, we found no increase for women aged less than 25 years (R = 0.46; aR^2^ = 0.11; *p* = 0.19), but, after 2007 (coincident with publication of the RANZCOG guidance), there were significant increases in the 25 to 34 year age group (R = 0.84; aR^2^ = 0.67; *p* = 0.002) and the 35 years and older age group (R = 0.99; aR^2^ = 0.96; *p* < 0.005) (Figure 3).

## 4. Discussion

This study of national trends in Australia demonstrates that, since the release of the RANZCOG guideline for the prevention of preterm birth in 2007, there has been a significant increase in the incidence rate of cervical cerclage in Australia, in women 25 years of age and older. This is a direct contrast to data from the United States which shows a fall in the use of cerclage [8]. Despite this increase and greater availability of progesterone vehicles, there has been no reduction in the incidence of early preterm birth in Australia.

Preterm birth occurs in about 6%–8% of all births in Australia and up to 10% of all pregnancies worldwide, with no evidence of any decrease in the rate in recent years [1,2,10]. Perinatal complications arising from preterm birth are well-recognised and include admission for neonatal intensive care, respiratory distress, requirement for mechanical ventilation, and childhood developmental impairment including cerebral palsy [1,2,11]. Perinatal complications of preterm birth have a high economic and social cost. Globally, there have been major efforts to reduce the burden of preterm labour and birth. The focus of such efforts largely have largely been directed toward identifying and, if possible, modifying the predominant risk factors for preterm birth: previous preterm birth; shortened cervix diagnosed on ultrasound examination; and, low socioeconomic status (which is linked to smoking and poorer general health, including low pre-pregnancy weight and minimal pregnancy weight gain).

Vaginally-placed cervical cerclage continues to be an intervention of choice in Australia. The Australasian Society of Ultrasound in Medicine (ASUM) recommends that cervical length should be assessed at the time of the mid-trimester morphology ultrasound [12]. At present, there is consensus that the median cervical length on transvaginal ultrasound at 22 to 25 weeks of gestation is 35 mm, and that 20 mm equates to the 5th centile [13,14]. There is evidence that intervention for cervical shortening found on the fetal anomaly ultrasound scan at 20 weeks is indicated to reduce the risk of preterm birth. Use of vaginally-placed cervical cerclage has increased in Australia for women 25 years and over, in the period 2007–2013.

Preterm birth is closely linked to cervical shortening; however, this shortening is likely to be one factor in an obstetric complex or syndrome that contributes to preterm labour and birth [11]. Cervical cerclage has been the traditional management technique used to reduce the incidence of preterm birth in women suspected of or being shown to have a shortened cervix, commonly on the basis of history and ultrasound examination in mid-trimester. Progesterone supplementation has now demonstrated benefit in this setting [11]. It has been surmised that the effectiveness of vaginal progesterone is due to a decline in innate progesterone in women at risk of preterm birth [11]. Vaginal progesterone supplementation has been demonstrated to effectively reduce the rate of preterm birth. One study demonstrated a reduction in preterm birth of as much as 44% [11]. Use of progesterone supplementation may explain why the incidence of cervical cerclage North America has reduced. Further research to directly compare the efficacy of cervical cerclage with that of vaginal progesterone would be helpful.

## 5. Conclusions

Prematurity is the single most important cause of perinatal mortality and morbidity. Women with documented shortening of cervix in mid trimester and those with a history of previous preterm delivery, recurrent cervical dilatation or previous cervical procedures are at particular risk of preterm labour. There is also consensus data to suggest women who have low progesterone levels are also at risk of preterm birth. Identifying women at risk of preterm delivery and working towards the prevention of preterm birth is topical in current international obstetric literature. Reducing the rate of preterm birth nationally and globally will significantly reduce social and economic costs. To date, no single strategy has been proven to have significant efficacy over another as the aetiology of preterm labour is often multifactorial.

Our study demonstrates that the use of cervical cerclage for the management of women at risk of preterm birth has increased in the study period (2007–2013) for women aged over 25 years. This is in a setting where the rates of preterm birth in Australia for babies 20–27 weeks and 28–31 weeks have actually increased, by a small but significant amount. This trend contrasts with the published trend in the US, which demonstrates a movement away from cerclage in favour of vaginal progesterone supplementation to reduce the rate of preterm birth. Cerclage has been the mainstay of management for women at risk of preterm birth for many years. In Australia the RANZCOG guideline supports the use of cerclage. With an awareness and counselling of the risks associated, cerclage clearly still has a place in practice, however in order to challenge and reverse the statistics, comparison between vaginal progesterone supplementation and cerclage would be helpful, in addition to further investigation of the pathophysiology and aetiology of preterm birth and its risk factors.
